# Explainable Multitask Burnout Prediction Using Adaptive Deep Learning (EMBRACE) for Resident Physicians: Algorithm Development and Validation Study

**DOI:** 10.2196/57025

**Published:** 2026-01-08

**Authors:** Saima Alam, Mohammad Arif Ul Alam

**Affiliations:** 1 Merrimack Health Methuen Hospital Methuen, MA United States; 2 Richard A. Miner School of Computer and Information Sciences University of Massachusetts Lowell Lowell, MA United States; 3 University of Massachusetts Chan Medical School Worcester, MA United States; 4 National Institute on Aging National Institute of Health Bethesda, MD United States

**Keywords:** future burnout prediction, wearable sensors, machine learning, multitask learning, clinical explainability, health care informatics

## Abstract

**Background:**

Medical residency is characterized by high stress, long working hours, and demanding schedules, leading to widespread burnout among resident physicians. Although wearable sensors and machine learning (ML) models hold promise for predicting burnout, their lack of clinical explainability often limits their utility in health care settings.

**Objective:**

This paper presents EMBRACE (Explainable Multitask Burnout Prediction Using Adaptive Deep Learning), a novel framework designed to predict and explain future burnout in resident physicians through an adaptive multitask deep learning approach. The framework aims to provide clinically actionable and trustworthy burnout predictions by integrating explainable ML techniques.

**Methods:**

EMBRACE applies deep multitask learning (3 tasks) using wearable sensor data for context-aware burnout prediction and explanation. The adaptive multitask learning framework predicts workplace activities and future burnout levels, and automatically completes a clinically validated burnout survey. Additionally, an explainability study was conducted using SHAP (Shapley Additive Explanations) to provide feature importance scores and visualizations for clinicians, enhancing the transparency and interpretability of the predictions. We evaluated the model on three datasets: (1) a collected dataset of 28 resident physicians (mean age 27.5, SD 3.5 years), over 2-7 days (average 3.6 days) with research protocols approved by the institutional review board (#2021-017) of Berkshire Medical Center, University of Massachusetts Chan Medical School; (2) the publicly available WESAD (Wearable Stress and Affect Detection) dataset from 15 participants; and (3) the SWELL-KW (SWELL Knowledge Work) dataset containing workplace stress and activity data from 25 participants (8 females and 17 males).

**Results:**

On our collected dataset, EMBRACE achieved 93% recall, 91% precision, and 0.91 *R*^2^ error in predicting 5-class activities, 4-class future burnout levels, and 1 clinically explainable survey (Mini-Z with 10 questions). On the WESAD dataset, the model achieved 94.1% recall and 94.6% precision for 3-class stress level prediction. On the SWELL-KW dataset, EMBRACE obtained 89% recall, 86% precision, and 0.88 *R*^2^ error in predicting 5-class activities, 3 burnout measures (joyful, satisfaction, and stress) with 2 classes on each measure, and 4 survey assessments (a total of 20 questions). The explainability study, using SHAP values, highlighted key contributing factors such as heart rate variability, sedentary activity duration, and interruptions, improving clinical trust and interpretation of burnout predictions. Of 23 participants, 21 (91%) reported satisfaction with the explainability of feature importance summaries.

**Conclusions:**

EMBRACE provides a clinically explainable and actionable solution for early burnout detection in resident physicians, leveraging advanced ML techniques and SHAP-based explanations. Validation of proprietary and publicly available datasets demonstrates their robustness and generalizability. Future research may explore scaling the model across different clinical environments and assessing its long-term impact on health care outcomes and physician well-being.

## Introduction

### Foundations of Physician Burnout

Burnout is a psychological syndrome emerging as a prolonged response to chronic interpersonal stressors on the job. It is characterized by 3 dimensions: emotional exhaustion, depersonalization, and reduced personal accomplishment. Stress, on the other hand, is a more immediate reaction to a challenge or demand, often leading to burnout when experienced frequently or intensely. In our work, we focus on predicting physician burnout by analyzing the stress levels observed through various wearable sensors.

### Background

Workplace stress is a pervasive issue that affects individuals across various professions and industries [1]. It encompasses the psychological, emotional, and physical strain experienced by employees due to demanding work conditions, excessive workload, and challenging interpersonal dynamics [2]. Recent statistics highlight the magnitude of the workplace stress problem, with studies indicating that 80% of employees reported feeling stressed at work sometimes, and 60% of absenteeism was associated with stress in some ways in that survey [3,4]. This alarming trend raises concerns about the impact of workplace stress on individuals’ well-being, job satisfaction, and overall quality of life [5].

Recognizing the detrimental effects of workplace stress, researchers and clinicians have developed clinically validated tools to assess and detect stress levels in workers [6]. These tools typically involve questionnaires and surveys that measure various dimensions of stress, including task load, mental effort, emotion, and perceived stress [7]. Additionally, real-time methods for quantifying continuous mental workload have been proposed [8]. One widely used tool is the Maslach Burnout Inventory, which evaluates burnout by measuring emotional exhaustion, depersonalization, and personal accomplishment among professionals [9]. Another prominent tool is the Copenhagen Burnout Inventory, which focuses on personal, work-related, and client-related burnout, providing a comprehensive view of burnout sources [10]. The Perceived Stress Scale is frequently used to measure the perception of stress in workers, assessing how unpredictable, uncontrollable, and overloaded respondents find their lives [2,11]. Additionally, the Job Content Questionnaire assesses job characteristics such as decision latitude, psychological demands, and social support at work, which are critical factors influencing stress and burnout [12]. The Mini-Z survey is another widely used tool that assesses various dimensions of burnout and job satisfaction, including stress, workload, and control over work, making it effective in both clinical and research settings [10,13]. These tools help in identifying stress levels and sources, allowing for targeted interventions to mitigate the adverse effects of workplace stress and improve overall well-being.

While these tools provide valuable insights and are clinically explainable to nurses and clinicians, they are often limited by their reliance on self-reporting and retrospective assessments, which can be subject to recall biases and may not capture real-time stress experiences [14]. To address these limitations and provide real-time monitoring of workplace stress, wearables and machine learning (ML) techniques have emerged as promising solutions. Wearable devices equipped with sensors can collect physiological and behavioral data from individuals throughout their workday, offering continuous and objective measurements of stress-related indicators such as heart rate variability, skin conductance, and physical activity. These devices have been extensively used in various studies to monitor and assess stress levels in real time. For instance, a study validated the Empatica E4 wristband’s ability to detect heart rate variability and electrodermal activity (EDA) metrics in stress-inducing conditions [15]. Another research project focused on the continuous monitoring of stress using photoplethysmogram sensors integrated into wrist-worn devices, highlighting significant changes in physiological responses during stress-inducing tasks [16]. These developments underscore the potential of wearable technology in providing reliable, objective, and continuous stress monitoring solutions [17]. ML algorithms can then analyze these data and predict stress levels in real time [18].

Medical residency is undeniably one of the most challenging and demanding workplace stress situations that individuals can experience. Medical residency is a highly challenging and demanding period characterized by extended working hours and schedules [19]. The demanding work schedules and long hours of residency, coupled with work-home interference, create a highly stressful environment that predisposes residents to burnout due to several stressors, including sleep deprivation, conflicts with coworkers, difficulty adapting to a new environment, heavy patient responsibilities, lack of control over schedules, and personal traits such as neuroticism or introversion that increase the risk of burnout [20]. Burnout can cause physical symptoms (headache, fatigue, gastrointestinal distress, flu, and sleep and appetite changes) and psychological symptoms (irritability and reduced concentration), as well as behaviors like procrastination, daydreaming, and substance use [21]. Additionally, it can lead to an increased risk of depression, suicidal thoughts, and cardiovascular problems [22]. Moreover, the COVID-19 pandemic has exacerbated the long-standing issue of resident burnout in the US health care system, highlighting the urgent need for interventions to support and protect the well-being of these essential frontline workers before it is too late [23]. The combined use of advanced wearable sensor technologies and ML algorithms can facilitate the early identification of burnout, thereby providing an opportunity to prevent its occurrence [18].

Despite their potential benefits, wearable sensors and ML-based predictions may suffer from a lack of clinical explainability, potentially leading to mistrust among clinicians and limiting their practical use in real-time clinical settings [24,25].

### Contributions

This paper introduces a novel framework, EMBRACE (Explainable Multitask Burnout Prediction Using Adaptive Deep Learning), for enhancing the prediction and explanation of future burnout in residents by using a clinically validated survey that is easily comprehensible and reliable for clinicians. More specifically, our key contributions are

- In EMBRACE, we develop a wearable sensor-based improved workplace activities and stress recognition framework using a deep multitask learning (MTL) technique. Then, using that, we develop a novel explainable MTL framework to automatically predict future burnout and explain the prediction by filling out a clinically validated and trustworthy burnout prediction survey tool.
- We validated the accuracy and explainability of our proposed EMBRACE framework using real-time collected data from 28 internal medicine residents (2-7 days each) in a natural hospital duty setting with appropriate institutional review board approval (#2021-017) of Berkshire Medical Center of the University of Massachusetts Chan Medical School.
- We assessed the generalizability of the EMBRACE framework by testing its performance on two publicly available occupational stress prediction datasets. The results demonstrated the framework’s robustness and effectiveness across diverse datasets, highlighting its potential for broader application in real-world settings.

### Related Work

#### ML Approaches to Burnout Detection

The use of ML techniques in detecting burnout among resident physicians is a relatively new area of research. While ecological momentary assessment has shown effectiveness in predicting burnout among residents [26], incorporating ML methods has the potential to enhance prediction performance [27]. However, real-time burnout prediction necessitates continuous monitoring of health vitals and ML techniques [28-30]. Recent systematic reviews [29,30] indicate that existing just-in-time burnout prediction techniques use biomarkers such as skin temperature, motion-based activities (accelerometers), electrodermal fluctuations, and wristband-based blood volume pulse. Various ML algorithms such as multilayer perceptron (MLP), random forest, *k*-nearest neighbors, support vector machine, linear regression, convolutional neural networks (CNN), fully convolutional network, Time-CNN, ResNet MLP, CNN-LSTM (long short-term memory), MLP-LSTM, InceptionTime, and others have been used in these studies [29,30]. However, a common limitation among these works is the lack of clinical explainability, which has not been adequately addressed in this research field [25,29,30].

#### Multitask Deep Learning Frameworks on Wearable Sensor Computing

Recent advancements in deep learning (MTL) frameworks have demonstrated significant improvements in the performance of wearable sensor computing. Taylor et al [31] developed an MTL model that simultaneously predicts physical activity levels and stress markers using data from wearable devices. Their approach highlighted the benefits of shared representations in improving the generalizability and accuracy of the predictions [31]. Similarly, Sabry et al [32] introduced a deep MTL framework for health monitoring that integrates tasks such as activity recognition, sleep stage detection, and stress level prediction, showing enhanced performance over single-task models. Another noteworthy contribution by Arefeen and Ghasemzadeh [33] focused on leveraging MTL to predict both physiological and behavioral responses, illustrating the model’s robustness across different wearable sensor datasets.

#### Context-Aware Stress Prediction Using Wearables

Context-aware stress prediction has gained traction as it enables more accurate and personalized stress monitoring. Aqajari et al [34] proposed a context-aware framework that uses environmental and physiological data from wearable sensors to predict stress levels, achieving higher accuracy compared to context-agnostic models. Similarly, Campana and Delmastro [35] developed a context-aware stress monitoring system that integrates location-based data and social interactions with physiological signals, demonstrating significant improvements in stress prediction accuracy. The work by Zhang et al [36] further advanced this field by incorporating ML algorithms to analyze multimodal sensor data, thereby providing real-time stress detection and feedback.

#### Explainable Wearable Sensor Computing

Many researchers proposed different interpretable and explainable artificial intelligence (AI) algorithms to make complex AI prediction models explainable, which include the Additive Feature Attribution method and the local interpretable model-agnostic explanations (LIME) approach [37]. The SHAP (Shapley Additive Explanations) approach combines LIME with Shapley values to provide explanations for black-box models [38]. Other methods include class activation mapping [39], DeepLIFT (Deep Learning Important Features) [40], and layer-wise relevance propagation [41] for interpreting CNNs. In health care, explainable AI applications have been developed for interpreting imaging studies and real-time predictions [42]. One previous work proposed interpretable ML techniques for stress prediction using wearables, but it only provided a simplistic representation of top features based on SHAP, which lacks clinical significance [43]. Adapa et al [44] proposed a supervised ML method to predict burnout among resident physicians that takes a bunch of surveys to understand different workplace problems and activities related to it, and—based on those longitudinal surveys on personal, physical, workplace environmental, and physiological status measures—performed a supervised ML approach to identify some highly correlated factors (emotional exhaustion, depersonalization, race demographics, etc). EMBRACE offers both efficient burnout prediction and a clinically validated survey-filling-out method, hypothesizing that the clinical survey of burnout estimation is explainable and trustworthy among resident physicians. Recent studies have focused on making these systems more interpretable. Abdelaal et al [45] introduced an explainable AI framework for wearable health monitoring that uses SHAP values to provide insights into model predictions, enhancing trust among clinicians. Additionally, De Cannière et al [46] proposed an interpretable deep learning model that visualizes feature importance and decision pathways, making the model’s outputs more comprehensible for end users. Another significant contribution by Kyriakou et al [47] involves the development of a transparent stress detection system that combines rule-based logic with ML to offer clear explanations of its predictions.

Our proposed EMBRACE framework leverages a clinically explainable, multitask adaptive deep learning approach, making it superior by providing trustworthy and actionable insights for burnout prediction. By integrating context-aware stress prediction with explainable AI techniques, EMBRACE ensures high accuracy and transparency. This combination addresses the limitations of existing models, thereby enhancing the practical utility of wearable sensor computing in clinical settings.

The primary aim of this study is to develop and validate the EMBRACE framework, a clinically explainable adaptive multitask deep learning model, for predicting and explaining future burnout among resident physicians using wearable sensor data. We hypothesize that integrating real-time physiological data, context-aware activity recognition, and explainable ML techniques will significantly enhance the accuracy, interpretability, and clinical trustworthiness of burnout predictions. We further hypothesize that the EMBRACE framework’s performance will generalize effectively across diverse clinical environments, supporting timely interventions to mitigate burnout and promote physician well-being.

## Methods

The EMBRACE framework consists of two core components: (1) an algorithm for detecting workplace activity and stress using a publicly available dataset and (2) an adaptive algorithm for detecting burnout level and explanation in our collected dataset, as well as in the publicly available dataset [3].

### Publicly Available Wearable Stress and Affect Detection Dataset (D1)

We used the WESAD (Wearable Stress and Affect Detection) public dataset [48]. This dataset comprises recordings from 15 participants (12 male and 3 female) who were equipped with 2 wearable devices: the RespiBAN Professional and the Empatica E4. The RespiBAN device, positioned on the chest, captured signals such as body acceleration (along 3 axes), body temperature, respiration, electrocardiography, electromyography, and EDA, all sampled at a frequency of 700 Hz. The Empatica E4 wristband measured signals including hand acceleration (along 3 axes), skin temperature, blood volume pulse, and EDA, with these signals being recorded at varying sampling rates. All signals from the Empatica E4 were subsequently upsampled to a uniform rate of 64 Hz using the Fourier method. The participants selected for this study excluded individuals with mental or cardiovascular conditions, those who were pregnant, and heavy smokers, with an average age of 27.5 years. During the data collection phases, participants either stood or sat during the baseline, amusement, and stress phases (with half of the participants standing and the other half sitting for each phase). In contrast, all participants sat during the meditation phase (for details, see Multimedia Appendix 1) [49-52].

Building upon previous research on stress detection using the WESAD dataset [48], we considered 3 distinct classification tasks in this study. The first task [48] focused on distinguishing between stress and nonstress states using data from 3 phases: baseline, stress, and amusement. The aim was to classify stress (stress phase) versus nonstress (baseline and amusement phases) (S vs NS). The second task [48] aimed to differentiate among 3 states: baseline, stress, and amusement (B vs S vs A). The third task [48] extended the classification to 5 distinct classes: baseline, stress, amusement, meditation, and recovery (B vs S vs A vs M vs R).

### Publicly Available Stress and User Modeling Dataset, SWELL-Knowledge Work Dataset (D2)

The SWELL-KW (SWELL Knowledge Work) dataset comprises accelerometer, heart rate, and galvanic skin response sensor data along with activity labels and subjective stress assessments from workplace activities [50-52]. Data were collected from 25 participants (average age 29, SD 4.2 years) performing tasks in controlled laboratory scenarios designed to induce stress (neutral, time pressure, and email interruptions). Each participant completed all scenarios over a 3-hour session, with sensors operating at 50 Hz (accelerometers), 1 Hz (heart rate monitors), and 10 Hz (galvanic skin response sensors). Activity labels included making presentations, paper writing and planning, writing and reading emails, programming, creating overviews, information searching, and time away from the keyboard, annotated via video recordings for accuracy.

Subjective stress was assessed using 4 validated surveys: NASA (National Aeronautics and Space Administration) Task Load Index (NASA-TLX), Rating Scale Mental Effort (RSME), Self-Assessment Manikin (SAM), and Perceived Stress Scale (PSS) [53]. NASA-TLX measures task load across mental, physical, and temporal demand, performance, effort, and frustration (scores are averaged, with higher scores indicating higher stress). RSME rates mental effort (0-150 scale; higher indicates higher stress). SAM captures valence, arousal, and dominance emotions pictorially (higher arousal and lower valence indicate higher stress) [54]. PSS provides a global measure of perceived stress (10-item, 0-40 scale; for details, see Multimedia Appendix 1) [53].

### Ethical Considerations

The study received approval (exemption) from the institutional review board (#2021-017) of Berkshire Medical Center of the University of Massachusetts Chan Medical School. Participants voluntarily participated in this study and provided informed consent before enrollment. All data were stored in a secure, HIPAA (Health Insurance Portability and Accountability Act)-compliant server with proper deidentification to protect participant privacy. The study adheres to ethical guidelines and regulatory requirements for conducting research with human participants. Participation in this study was entirely voluntary. No incentives or gifts were provided to participants, a fact that was clearly communicated during recruitment and outlined in the consent document.

### Our Data Collection Principles

#### Medical and Clinical Tasks of Interest

The medical and clinical task of interest in our study is prognostic, focusing on predicting the future occurrence of burnout among internal medicine resident physicians. This involves continuous monitoring of physiological data using wearable sensors to estimate the risk of burnout, thereby allowing timely interventions.

#### Research Question

The primary research question addressed in this study is, “Can continuous monitoring of physiological data using wearable sensors, combined with ML techniques, accurately predict future burnout levels in resident physicians?” The outcomes of interest include the levels of burnout, stress, and satisfaction, as measured by the Mini-Z Burnout Survey [13]. The study aims to identify significant predictors of burnout and develop an explainable ML model to enhance clinical decision-making. The Mini-Z survey is widely recognized as a clinically validated and concise tool for assessing burnout, stress, and job satisfaction, making it ideal for our target study on resident physicians who face high-pressure environments. Its simplicity and focus on actionable dimensions like workload, electronic medical record (EMR) stress, and control over work ensure that it captures relevant factors contributing to burnout, aligning perfectly with the predictive goals of our EMBRACE framework. The survey’s structured 10-item format facilitates automated completion via ML models, enabling seamless integration with wearable sensor data for real-time burnout prediction. Mini-Z’s broad adoption in health care settings ensures that its results are interpretable and trustworthy for clinicians, enhancing the explainability and clinical utility of our framework. By targeting key predictors of burnout and providing clear thresholds for intervention, the Mini-Z survey supports our objective of delivering clinically actionable insights to improve resident physicians’ well-being.

#### Known Predictors and Confounders to What Is Being Predicted or Diagnosed

Predictors of burnout in this study include physiological measures such as heart rate variability, skin conductance, and physical activity levels, collected using the Empatica E4 watch [55]. These predictors are chosen based on existing literature that links them to stress and burnout. Confounders may include individual differences in baseline stress levels, workload intensity, and personal coping mechanisms. These factors are controlled through initial baseline assessments and continuous monitoring.

#### Overall Study Design

The study uses a prospective cohort design, where 28 internal medicine resident physicians are monitored over a period ranging from 2 to 7 days. Data collected includes physiological metrics from wearable sensors and responses to the Mini-Z Burnout Survey [13]. The study is divided into training, validation, and testing phases to develop and evaluate the ML model.

#### Medical Institutional Settings

The study is conducted at a renowned teaching-based medical center, Berkshire Medical Center of the University of Massachusetts Chan Medical School, where the internal medicine residency program is hosted. The collected data and the ML model are intended to be used in this clinical setting to monitor and predict burnout among resident physicians.

#### Target Population

This study targets internal medicine resident physicians from various postgraduate year (PGY1, PGY2, and PGY3) levels. The model aims to generalize across this population to provide accurate burnout predictions for different stages of residency training.

#### Intended Use of the ML Model

The ML model is intended to be used as a tool for continuous monitoring and early detection of burnout among resident physicians. It will provide real-time alerts to medical staff and wellness coordinators, enabling proactive interventions. The intended users (with residents’ consent) include clinicians, residency program directors, and wellness coordinators, who will use the model’s outputs to support residents’ well-being.

#### Existing Model Performance Benchmarks for This Task

Existing benchmarks for burnout prediction models typically involve metrics such as accuracy, recall, precision, and the area under the receiver operating characteristic curve. Previous studies using ML methods have reported varied performance, often limited by a lack of real-time data and clinical explainability. Our study aims to surpass these benchmarks by incorporating continuous physiological monitoring and explainable AI techniques.

#### Burnout Classes

Burnout levels were assessed using the Mini-Z Burnout Survey, which includes 10 questions scored on a 5-point Likert scale, along with an additional open-ended question. Three different burnout scales were derived from these responses:

1. Joyful Measure: The total score is calculated by summing the points from all 10 items, with a score range of 10 to 40 points. A score of 20 or higher indicates a joyful work environment, which has been used to design a 2-class problem: joyful or not joyful work environment.
2. Satisfaction Scale: This scale is derived by adding the points from questions 1, 2, 3, and 4, resulting in a score range of 4 to 25 points. A score of 20 or higher indicates a highly supportive environment, which has been used to design a 2-class problem: satisfied or not satisfied work environment.
3. Stress Scale: The stress scale is calculated by summing the points from questions 5, 6, 7, and 8, with a score range of 4 to 25 points. A score of 20 or higher indicates a low-stress environment with reasonable EMR pressures, which has been used to design a 2-class problem: high or low stress at work environment.

Participants were asked to complete the Mini-Z survey daily, and their responses were used to establish baseline burnout levels and track changes over the study period. This continuous assessment allows for timely interventions to prevent and mitigate burnout.

### Our Collected EMBRACE Dataset Description (D3)

The study included 28 internal medicine resident physicians (average age 27.5, SD 3.5 years) from a renowned teaching-based medical center, spanning different postgraduate years (PGY1, PGY2, and PGY3). Inclusion criteria required participants to be actively engaged in their residency program, while exclusion criteria involved any medical conditions that could interfere with stress and burnout assessment. Data collection was prospective, with participants wearing an Empatica E4 watch continuously from the start to the end of their daily duties, covering periods ranging from 2 to 7 days. Each participant contributed to a total of 98 days of data, with each day spanning 8 to 13 hours of working hours, averaging 10.5 hours per day, resulting in approximately 1029 hours of physiological data and 98 different daily ground truth data from surveys. Of 98 days, 33 (34%) were identified as burnout days (the days that ended with a burnout as per the burnout survey), spanning over 19 out of 28 (68%) residents. The collected data included heart rate variability, skin conductance, and physical activity levels, recorded at frequencies of 1, 10, and 50 Hz, respectively. Additionally, participants completed the Mini-Z Burnout Survey daily via a web-based form sent to their cell phones, providing subjective assessments of burnout, stress, and satisfaction. Potential biases include self-reporting inaccuracies and the variability in daily workloads, which were controlled through baseline assessments and continuous monitoring. The dataset consists of longitudinal records with multiple data points per participant, encompassing continuous (physiological measures) and categorical (survey responses) data. Data preprocessing involved normalizing physiological measures and handling missing data through imputation methods. Known quality issues include potential sensor malfunctions and variability in self-reported data. The sample size was deemed sufficient based on standard ML training requirements, ensuring adequate model performance and stability. The data are stored in a secured, HIPAA-compliant server and are available for further research upon request, adhering to data sharing policies. Table S1 in Multimedia Appendix 1 presents the description of the study.

### Detecting Workplace Activity and Stress Using Existing Dataset

#### Multitask Deep Learning for Joint Activity and Stress Detection

A multitask deep learning framework for wearable sensor-based activity and stress detection involves training a single model to simultaneously perform multiple tasks, specifically activity recognition and stress level classification. The framework combines both tasks into a single neural network architecture, allowing shared representations to be learned and leveraging the complementary information present in the data.

#### Input Data

The input data consist of time-series sensor readings from wearable devices, denoted as *X* ∈ *R^T^*^×^*^N^*, where *T* represents the length of the time series and *N* is the number of sensor channels.

#### Activity Recognition Task

Activity recognition aims to predict the activity type based on sensor data. The predicted activity labels are denoted as *Y*_act_ ∈ {0, 1}*^C^*_act_, where *C*_act_ represents the number of activity classes. The output layer for activity recognition is defined as

*O*_act_ = softmax(*W*_act_ × *H* + *b*_act_) (1)

where *H* represents the shared hidden representations obtained from the network, *W*_act_ is the weight matrix, and *b*_act_ is the bias term specific to the activity recognition task.

#### Stress Level Classification Task

Stress level classification aims to predict the stress level based on sensor data. The predicted stress labels are denoted as *Y*_stress_ ∈ {0, 1}*^C^*_stress_, where *C*_stress_ represents the number of stress level classes. The output layer for stress level classification is defined as

*O*_stress_ = softmax(*W*_stress_ × *H* + *b*_stress_) (2)

where *H* represents the shared hidden representations obtained from the network, *W*_stress_ is the weight matrix, and *b*_stress_ is the bias term specific to the stress level classification task.

#### Shared Representation Learning

The shared representation learning module learns a representation that captures both activity and stress-related patterns in the input data. This module consists of a combination of 1 CNN with 32 hidden nodes each and 2 LSTM layers with 64 hidden nodes each to extract meaningful features from the input time series. The final fused hidden representation obtained from this module is denoted as *H*.

#### Loss Function

The multitask loss function combines the losses from both tasks to jointly optimize the model. The loss function is defined as a combination of activity recognition loss (*L*_act_) and stress level classification loss (*L*_stress_), weighted by respective task-specific coefficients (*α* and *β*):

Loss = *α* × *L*_act_ + *β* × *L*_stress_ (3)

#### Learning

The model is trained using backpropagation and gradient descent optimization techniques, minimizing the multitask loss function. The shared representation learning module and task-specific layers are updated jointly during training. By training the multitask deep learning framework, the model learns to extract relevant features from the wearable sensor data and simultaneously perform activity recognition and stress level classification tasks. This joint learning approach enables the model to leverage the shared representations and potentially improve the performance of both tasks compared to training separate models.

### Burnout Prediction and Explanation

#### Multitask Few-Shot Domain Adaptation for Mini-Z Survey and Burnout Prediction

To build a multitask few-shot deep domain adaptation framework based on the previous framework, we will adapt it to the scenario where wearable sensor data serves as input, the source domain involves multitask stress and activity recognition, and the target domain focuses on predicting the answers to a multitask Mini-Z survey questionnaire [13] and burnout prediction. The objective is to estimate the overall burnout scale class based on the Mini-Z survey questions’ answers. We describe this model as follows.

#### Preliminaries

In this framework, we have a similar input data representation where the source domain framework is the previously described multitask deep learning architecture for stress and activity recognition tasks. The model architecture includes shared representation learning, output layers for activity recognition (*O*_act_) and stress level classification (*O*_stress_), and corresponding labels *Y*_act_ and *Y*_stress_. In the target domain, the focus shifts to predicting the answers to the multitask Mini-Z survey questionnaire. The objective is to estimate the overall burnout scale class based on the answers to the Mini-Z survey questions. For each Mini-Z survey question, a separate output layer is defined in the neural network architecture. The output layer for predicting the answer to question *i* is denoted as *O_i_* = *f*(*W_i_H* + *b_i_*), where *H* represents the shared hidden representations obtained from the network, *W_i_* is the weight matrix specific to question *i*, *b_i_* is the bias term associated with question *i*, and *f* is an appropriate activation function. The estimated overall burnout scale class is derived from the answers to the Mini-Z survey questions. This has been achieved by defining a range of total Mini-Z survey questions’ answers and mapping them to specific burnout scale classes.

#### Multitask Adaptive Loss Function

The multitask loss function for the target domain includes the task-specific loss for Mini-Z survey questions prediction (*L*_Mini-Z_) and the overall burnout scale class loss (*L*_burnout_), weighted by respective task-specific coefficients (*γ* and *δ*). The loss function is defined as

Loss = *γ* · *L*_Mini-Z_ + *δ* · *L*_burnout_ (4)

where *L*_burnout_ is the cross-entropy loss for the overall burnout scale class estimation, and *L*_Mini-Z_ is the *R*^2^ loss metric. *R*^2^ is a goodness-of-fit measure for regression models. This statistic indicates the percentage of the variance in the dependent variable that the independent variables explain collectively. *R*^2^ measures the strength of the relationship between our model and the dependent variable on a convenient 0%-100% scale (see Multimedia Appendix 1).

#### Few-Shot Domain Adaptation

Few-shot domain adaptation aims to transfer knowledge from the source domain to the target domain, even when labeled data in the target domain is limited [56]. We modify the Model-Agnostic Meta-Learning (MAML) algorithm [57] according to our multitask source and target problem, which allows the model to quickly adapt to new tasks using 10 labeled samples from each class. The modified MAML algorithm includes initialization of model parameters and source domain training. Then, the few-shot domain adaptation includes selecting a few target samples with labels to define a new target task with the cloned source model’s parameters. Then, for each target domain task, we perform a few gradient update steps on target parameters using few samples and compute the task-specific target loss in the inner loop; and compute the gradient of the task-specific target loss with respect to source parameters and update it. Finally, we evaluate the adapted target task model using Mini-Z survey answer–based prediction (see Algorithm S1 in Multimedia Appendix 1).

## Results

### Setup

#### Source and Target Dataset Setup

The EMBRACE burnout dataset (D3) we collected does not include ground truth data for activity recognition. However, to effectively interpret burnout, it is crucial to predict workplace activity summaries, evaluate burnout levels, and use clinically validated survey tools to enhance explainability and build trust among physicians. To address this, we used the SWELL-KW (D2) dataset as our source data. This dataset uses the same wearable sensor (Empatica E4) as ours and provides labeled workplace activities along with ground truth data for workplace stress assessment. In our problem setup, the target dataset is our collected EMBRACE dataset (D3).

#### Task Definitions

**Figure 1:**
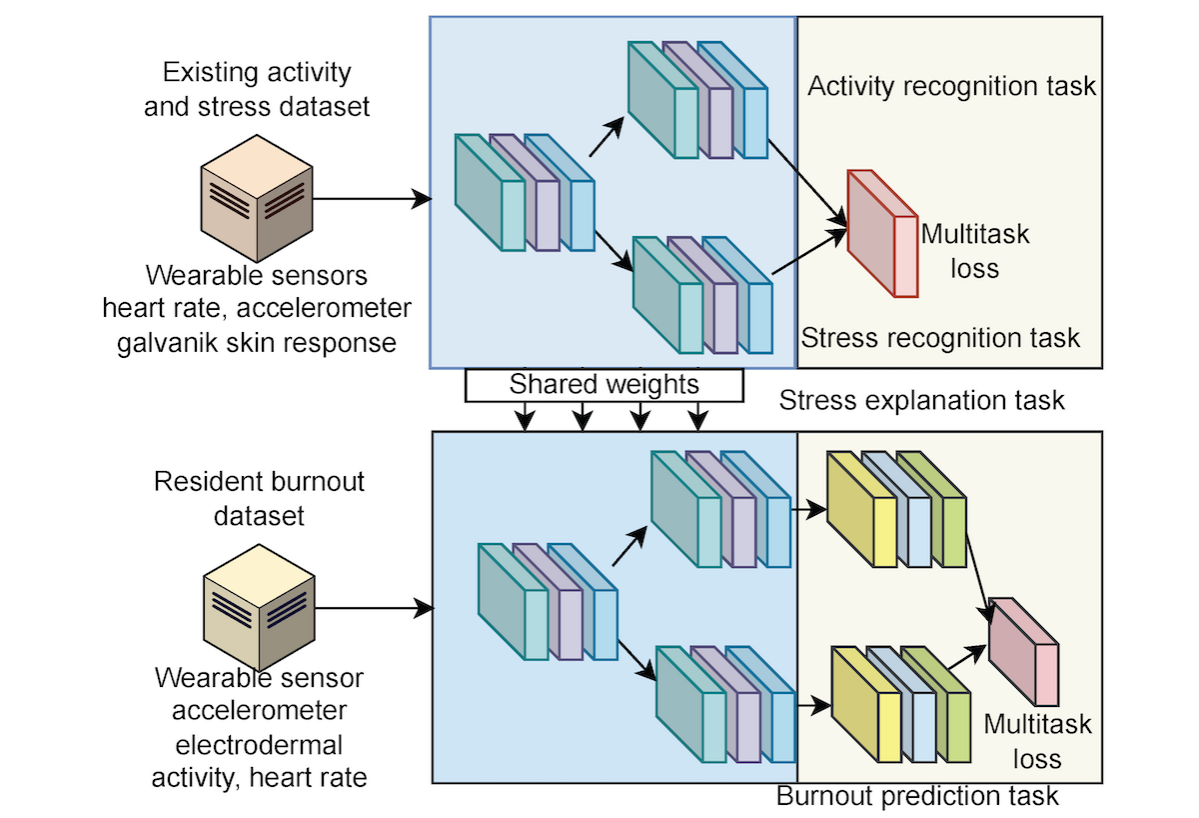
The schematic diagram of the proposed framework.

There are two tasks involved in the source dataset (D2)—task 1 (*T*_act_): 5-class activity recognition (writing reports, making presentations, reading email, searching for information, and others); and task 2 (*T*_stress_): 3-class stress level recognition (neutral, interruption, and time pressure). On the other hand, there are four tasks involved in the target dataset (D3)—task 1 (*T*_survey_answers_): a 10-class regression problem to fill out survey questions; task 2 (*T*_burnout1_): a 2-class overall measure (joyful work environment or not); task 3 (*T*_burnout2_): a 2-class satisfaction scale (highly supportive work environment or not); and task 4 (*T*_burnout3_): a stress scale (low stress environment with reasonable EMR pressure or not). In Figure 1, we present the schematic diagram of our entire framework with multiple task specifications.

#### Implementation

Our proposed model was implemented using Python’s Keras library with the TensorFlow backend. For the regression task, denoted as *T*_survey_answers_, we used the RMSE loss function. In contrast, for the classification tasks, which encompassed the remaining tasks, we used categorical cross-entropy loss. These loss functions were used while jointly training the few-shot MAML algorithm.

#### Hyperparameter Tuning

The optimization of our system was performed using the Adam optimization function with a learning rate of 1×10^−3^. The selection of the optimized learning rate and the weighting parameter *β* (set to 0.25) was achieved through hyperparameter tuning. The learning model of our framework was executed on a server equipped with a cluster of 3 Nvidia GTX GeForce Titan X GPUs and an Intel Xeon CPU (2.00 GHz) processor, along with 12 gigabytes of RAM.

#### Training

For training the multitask stress and workplace activity recognition framework, we used the D2 dataset (SWELL-KW) as input. This dataset included readings from wearable sensors such as accelerometers, heart rate monitors, and galvanic skin response sensors. The framework was trained to address two tasks. To adapt the shared module of the target adaptive multitask explainable burnout prediction, we used the trained weights for initialization (domain adaptation). Subsequently, we replaced the inputs with our collected dataset, D3, with readings from wearable sensors such as accelerometers, heart rate monitors, and EDA sensors. Additionally, we modified the output layer to accommodate the 4 aforementioned task problems.

#### Timeseries Leave-One-Out-Cross-Validation Setup

**Figure 2:**
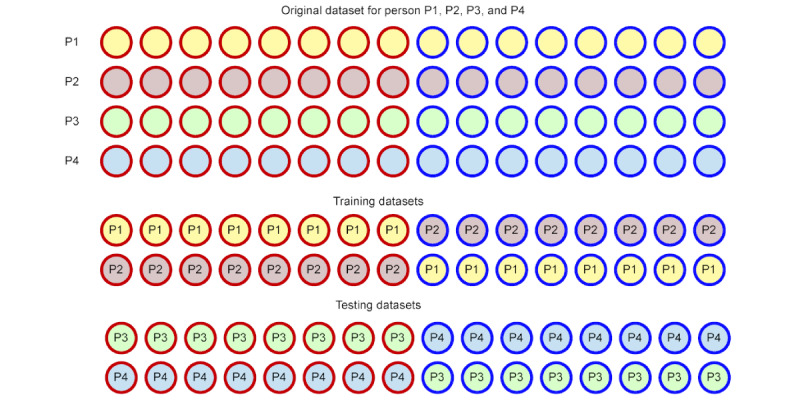
Example leave-one-person-out strategy-based training and testing sample generation without data leakage.

The conventional 10-fold cross-validation approach [58] is not suitable for sequential data. Therefore, to train and assess the performance of our proposed EMBRACE framework, we adopt a time-series cross-validation method [8,59]. Here, we partition the entire sequential dataset into two halves. Subsequently, we randomly select a sequence of data from the first half as the training sample and another random sequence from the second half as the testing sample. This process is repeated 10 times to generate 10 distinct pairs of training and testing data sequences. While generating such training and testing data sequences, we maintained a leave-one-person-out (leave-one-out cross-validation or LOOCV) strategy (leaving the training dataset included the individual relevant dataset out while selecting the testing dataset); thus, the person (out of 28) we chose to include in the training dataset would never be selected for the testing dataset. Figure 2 presents a sample of the LOOCV-based training and testing dataset generation technique that prevents data leakage between training and testing datasets.

#### Accuracy Evaluation Criteria

To evaluate individual task-level classification performance in the multitask setting of the EMBRACE framework, the accuracy metric was measured in a macro or balanced setting. For example, balanced accuracy calculates the accuracy for each task individually and then takes the average of these accuracies across all tasks, treating each task equally regardless of its sample size, using balanced accuracy (see Equations in Multimedia Appendix 1). This ensures a balanced contribution from all tasks to the overall performance metric. Balanced accuracy is suitable in scenarios where all tasks are equally important, and their performance needs to be evaluated independently of dataset size. It is particularly useful in MTL problems where sample sizes vary significantly between tasks.

To add more significance in the performance evaluation, we included balanced precision, recall, and *F*_1_-score as metrics too [60]. Additionally, we calculate the standard deviation of all these metrics to evaluate the presence of overfitting (Table 1).

**Table 1:** EMBRACE^a^ framework predicted individual Mini-Z burnout survey questionnaire–specific answers and overall burnout assessment performance (R2 coefficient), regression precision, recall, and F1-score stated in the Accuracy Evaluation Criteria section. Data are presented as mean% (SD%).

Questions	*R^2^*	Precision	Recall	*F*_1_-score
Q1	78.5 (0.9)	79.5 (0.9)	78.9 (0.8)	80.6 (0.9)
Q2	75.8 (0.7)	77.4 (0.8)	75.3 (0.9)	76.4 (0.9)
Q3	69.5 (1.9)	70.6 (1.1)	70.5 (1.2)	71.6 (1.0)
Q4	84.6 (0.9)	87.8 (0.7)	84.6 (0.9)	86.5 (0.9)
Q5	97.5 (0.01)	98.2 (0.01)	97.5 (0.01)	98.3 (0.01)
Q6	96.3 (0.01)	95.9 (0.02)	96.3 (0.01)	97.1 (0.02)
Q7	93.6 (0.02)	94.8 (0.03)	93.6 (0.02)	93.6 (0.01)
Q8	90.5 (0.3)	88.5 (1.1)	90.4 (0.2)	91.3 (0.8)
Q9	86.5 (0.9)	87.1 (1.2)	85.9 (0.5)	88.8 (0.9)
Q10	90.2 (1.0)	89.4 (1.1)	90.2 (1.0)	91.5 (0.8)
Overall	87.7 (0.5)	88.3 (0.8)	87.6 (0.4)	88.8 (0.7)

^a^EMBRACE: Explainable Multitask Burnout Prediction Using Adaptive Deep Learning.

To evaluate individual task-level regression performance (ie, the prediction explanatory power), we used *R*^2^ coefficient as the primary evaluation metric. *R*^2^ is a goodness-of-fit measure for regression models. This statistic indicates the percentage of the variance in the dependent variable that the independent variables explain collectively. *R*^2^ measures the strength of the relationship between your model and the dependent variable on a convenient 0%-100% scale. The percentage of *R*^2^ has been presented in Multimedia Appendix 1. For perfect prediction, *R*^2^=100, while *R*^2^=0 indicates no explanatory power. To estimate precision, recall, and *F*_1_-score for regression tasks, we discretized the regression into predictions by considering proximity between predicted and true values using a threshold value of *δ*=0.5.

### Workplace Activity Recognition Performance

The SWELL-KW (D2) dataset contains detailed annotations of several workplace activities for 25 participants, including activities such as making presentations, paper writing, paper planning, writing emails, reading emails, programming, creating overviews, searching for information, and being away from the keyboard. However, due to significant overlaps between some of these activities, it was challenging to accurately distinguish them using wearable accelerometers and EDA sensors alone. Therefore, we consolidated these activities into five distinct categories: (1) writing (paper writing and paper planning), (2) presenting (making presentations, programming, and creating overviews), (3) email (writing emails and reading emails), (4) searching (searching for information), and (5) others (time away from keyboard, etc).

Table 2 presents the overall accuracy, precision, recall, and *F*_1_-score for workplace activity recognition, with values of 91.6%, 93.1%, 91.6%, and 93.9%, respectively. These results are accompanied by reasonably low standard deviations, indicating no signs of overfitting. Notably, the classification of writing activities achieves a significantly higher accuracy of 97% compared to other tasks. To compare the performance of our activity recognition task, we implemented the Bi-LSTM (bidirectional long short-term memory) [61], perceptron [62], BayesNet [62], decision tree [62], and K-Star [62] algorithms. Table 2 presents a comparison of various performance metrics between our model and the baseline algorithms. The results demonstrate that our model outperforms all the baseline algorithms implemented in this study.

**Table 2:** Comparison of workplace activity recognition performance across different algorithms with the EMBRACE^a^ framework. Data are presented as mean% (SD%).

Algorithms	Accuracy	Precision	Recall	*F*_1_-score
K-Star	76.4 (1.5)	75.8 (1.6)	76.4 (1.5)	77.2 (1.7)
Decision tree	80.2 (1.2)	81.5 (1.3)	80.2 (1.2)	81.9 (1.5)
BayesNet	82.9 (1.1)	83.1 (1.0)	82.9 (1.1)	84.0 (1.2)
Perceptron	86.5 (1.0)	86.9 (1.1)	86.5 (1.0)	87.4 (1.0)
Bi-LSTM^b^	91.4 (1.0)	93.0 (0.6)	91.4 (1.0)	93.7 (0.4)
Ours	91.6 (0.9)	93.1 (0.5)	91.6 (0.9)	93.9 (0.2)

^a^EMBRACE: Explainable Multitask Burnout Prediction Using Adaptive Deep Learning.

^b^Bi-LSTM: bidirectional long short-term memory.

### Stress Classification Performance

#### Linking Stress to Burnout and Use of Existing Datasets

Stress and burnout are closely linked, with chronic stress being a significant predictor of burnout in many occupations. Prolonged exposure to stress without sufficient recovery leads to emotional exhaustion, one of the key components of burnout [9]. Research has shown that stress affects not only physical health but also cognitive and emotional functioning, contributing to higher rates of burnout in high-demand environments [63]. Additionally, the accumulation of stress over time without effective coping mechanisms has been associated with an increase in depersonalization and reduced personal accomplishment, further solidifying the connection between stress and burnout [64]. Since wearable sensor-based burnout prediction datasets are not available, we apply our proposed framework to existing wearable stress datasets, such as the WESAD (D1) [48] and SWELL-KW (D2) [50-52] datasets.

#### WESAD Data

The WESAD (D1) dataset includes 5 emotional states: baseline, amusement, stress, meditation, and recovery. However, the WESAD researchers noted that meditation and recovery are not typical everyday emotional states and focused on the 3 primary states: baseline, amusement, and stress [48]. Following their approach, we excluded all data related to the meditation and recovery states, reducing the dataset to a 3-class problem. Table 3 reports the overall accuracy, precision, recall, and *F*_1_-score for stress level recognition on the WESAD (D1) dataset, with values of 94.1%, 94.2%, 94.1%, and 94.6%, respectively. Similar to the activity recognition results, the standard deviations remain reasonably low, indicating no signs of overfitting. Notably, the classification of the baseline stress level achieves an impressive accuracy of 98.9%. To compare with existing algorithms, we implemented SELF-CARE [65], the Gaussian mixture model, and CNN algorithms (Table 4). The SELF-CARE method uses selective sensor fusion and context-aware techniques to enhance stress detection accuracy, achieving an accuracy of 86.34%, a precision of 87.2%, a recall of 85.9%, and an *F*_1_-score of 86% for 3-class stress classification [65].

**Table 3:** Proposed algorithm-based 3-class stress level (baseline, stress, and amusement) classification performance details on the publicly available WESADa (D1) dataset. Data are presented as mean% (SD%).

Stress levels	Accuracy	Precision	Recall	*F*_1_-score
Baseline	98.9 (0.01)	97.8 (0.02)	98.9 (0.01)	98.6 (0.02)
Stress	93.7 (0.08)	94.8 (0.02)	93.7 (0.08)	95.5 (0.07)
Amusement	90.8 (0.10)	91.9 (0.10)	90.8 (0.10)	92.0 (0.09)
Overall	94.1 (0.03)	94.2 (0.03)	94.1 (0.03)	94.6 (0.02)

^a^WESAD: Wearable Stress and Affect Detection.

**Table 4:** Comparison of the proposed algorithm with state-of-the-art algorithms on the WESAD^a^ (D1) dataset to predict 3-class stress levels (baseline, stress, and amusement). Data are presented as mean% (SD%).

Algorithms	Accuracy	Precision	Recall	*F*_1_-score
Gaussian mixture model [48]	82.5 (1.2)	83.2 (1.1)	82.5 (1.2)	84.0 (1.3)
Convolutional neural networks [48]	89.8 (0.9)	90.5 (1.0)	89.8 (0.9)	90.7 (0.8)
Random forest [48]	86.2 (1.0)	87.0 (0.8)	86.2 (1.0)	87.4 (0.7)
SELF-CARE [65]	86.34 (0.8)	87.2 (0.6)	85.9 (0.7)	86.0 (0.6)
Ours	91.6 (0.9)	93.1 (0.5)	91.6 (0.9)	93.9 (0.2)

^a^WESAD: Wearable Stress and Affect Detection.

#### SWELL-KW Data

The SWELL-KW (D2) dataset contains stress data collected from participants under 3 work conditions: neutral, interruptions, and time pressure. Table 5 reports the overall accuracy, precision, recall, and *F*_1_-score performance metrics of our proposed algorithm for 3-class stress level classification on the SWELL-KW (D2) dataset, with values of 94.7%, 94.7%, 94.7%, and 95.1%, respectively. Similar to the results from the WESAD dataset, the standard deviations remain low, indicating no signs of overfitting. Notably, the classification of the neutral stress level achieves an impressive accuracy of 99.5%.

**Table 5:** Proposed algorithm-based 3-class stress level (neutral, interruptions, and time-pressure) classification performance details on the publicly available SWELL-KWa (D2) dataset. Data are presented as mean% (SD%).

Stress levels	Accuracy	Precision	Recall	*F*_1_-score
Neutral	99.5 (0.0)	98.2 (0.01)	99.5 (0.0)	99.1 (0.01)
Interrupt	94.1 (0.07)	95.4 (0.01)	94.1 (0.07)	96.3 (0.06)
Time	91.2 (0.09)	92.7 (0.09)	91.2 (0.09)	92.8 (0.08)
Overall	94.7 (0.02)	94.7 (0.02)	94.7 (0.02)	95.1 (0.01)

^a^SWELL-KW: SWELL Knowledge Work.

To compare with existing algorithms, we implemented the following models stated in Table 6. Koldijk et al [66] used the SWELL-KW dataset and compared several ML algorithms. Support vector machine with an radial basis function kernel achieved an accuracy of 90.03%, while other models like Naive Bayes, K-Star, and BayesNet achieved lower accuracies of 64.77%, 65.81%, and 69.08%, respectively. More advanced models like random forest (87.09%) and MLP (88.54%) outperformed simpler methods [66]. Similarly, de Vries et al [67] used a learning vector quantization approach, achieving 88% accuracy for stress classification. Based on these results, we can conclude that our framework demonstrates competitive performance against other existing methods.

**Table 6:** Comparison of the proposed algorithm with state-of-the-art algorithms on the SWELL-KW^a^ (D2) dataset to predict 3-class stress levels (neutral, interruptions, and time-pressure). Data are presented as mean% (SD%).

Algorithms	Accuracy	Precision	Recall	*F*_1_-score
Naive Bayes	64.77 (4.3)	69.56 (3.9)	66.89 (2.5)	67.45 (3.5)
K-Star	65.81 (3.8)	63.8 (3.7)	67.53 (4.1)	66.72 (4.1)
BayesNet	69.08 (2.5)	70.0 (3.1)	70.1 (1.9)	69.08 (2.1)
Support vector machine (RBF^b^ kernel) [66]	90.03 (0.8)	90.1 (0.7)	90.03 (0.8)	91.0 (0.9)
Random forest [66]	87.09 (1.0)	87.7 (0.9)	87.09 (1.0)	87.5 (1.1)
Multilayer perceptron [66]	88.54 (1.2)	89.3 (1.1)	88.54 (1.2)	89.1 (1.3)
Learning vector quantization [67]	88.0 (1.1)	88.5 (0.9)	88.0 (1.1)	88.4 (0.8)
Ours	94.7 (0.9)	94.7 (0.5)	94.7 (0.9)	95.1 (0.2)

^a^SWELL-KW: SWELL Knowledge Work.

^b^RBF: radial basis function.

#### EMBRACE Dataset

The EMBRACE dataset contains data for predicting burnout levels based on several measures, including the joyful measure, satisfaction scale, and stress scale. In addition to burnout measures prediction, we also use Mini-Z survey questions to predict specific responses for questionnaire completion. Tables 7 and 8 present the regression and classification performance for survey question completion and burnout prediction using our adaptive MTL framework.

**Table 7:** EMBRACE^a^ framework–based burnout prediction performance details on our collected dataset. Note that the Mini-Z burnout survey has 3 burnout measures (joyful measure, satisfaction scale, and stress scale) with 2 classes each to classify. Data are presented as mean% (SD%).

Burnout measures	Accuracy	Precision	Recall	*F*_1_-score
Joyful measure	82.7 (0.1)	83.5 (0.2)	82.5 (0.15)	81.3 (0.14)
Satisfaction scale	79.2 (0.1)	80.5 (0.2)	78.4 (0.15)	79.5 (0.2)
Stress scale	89.3 (0.05)	87.6 (0.11)	89.5 (0.1)	90.3 (0.1)
Overall	85.1 (0.1)	86.4 (0.1)	84.8 (0.2)	86 (0.1)

^a^EMBRACE: Explainable Multitask Burnout Prediction Using Adaptive Deep Learning.

**Table 8:** Comparison of Mini-Z survey questionnaire–specific answer score (regression problem) prediction performance of our proposed algorithm with state-of-the-art algorithms, where individual answer ranges from 1 to 5. Data are presented as mean% (SD%).

Algorithms	*R* ^2^	Precision	Recall	*F*_1_-score
Random forest [66]	82.6 (1.0)	82.8 (0.9)	82.3 (1.1)	83.2 (1.0)
Decision tree [68]	80.3 (1.1)	80.6 (0.9)	79.8 (1.2)	81.0 (0.8)
Bi-LSTM^a^ [61]	85.7 (0.8)	86.1 (0.7)	85.4 (0.9)	86.5 (0.8)
Ours	87.7 (0.5)	88.3 (0.8)	87.6 (0.4)	88.8 (0.7)

^a^Bi-LSTM: bidirectional long short-term memory.

Table 1 shows that our framework performs well in predicting survey question responses, with overall percentage *R*^2^ coefficient, precision, recall, and *F*_1_-score of 87.7%, 88.3%, 87.6%, and 88.8%, respectively (refer to the Accuracy Evaluation Criteria section). Although a few questions (such as Q1, Q2, and Q3) show relatively lower performance, the adaptive MTL framework efficiently compensates, yielding robust overall results.

Table 8 shows that our EMBRACE framework outperforms several baseline algorithms, including random forest, decision tree, and Bi-LSTM, in predicting Mini-Z survey questionnaire responses. With an overall percentage *R*^2^ coefficient, precision, recall, and *F*_1_-score of 87.7%, 88.3%, 87.6%, and 88.8%, respectively, the framework demonstrates robust performance. Notably, while some questions (eg, Q1, Q2, and Q3) exhibit lower individual performance, the adaptive MTL approach effectively compensates for these discrepancies, ensuring reliable overall results. Compared to other models, EMBRACE achieves higher precision and recall across all metrics, highlighting its superior ability to capture the nuances of physician burnout through clinically validated survey responses.

Table 9 reports the performance for burnout prediction, achieving an overall balanced accuracy, precision, recall, and *F*_1_-score of 94.7%, 94.7%, 94.7%, and 95.1%, respectively (refer to the Accuracy Evaluation Criteria section). The standard deviations across both tasks remain low, indicating no signs of overfitting.

To compare with existing algorithms, we implemented learning vector quantization, random forest, and Bi-LSTM [61], all of which have been shown to perform well in burnout and stress prediction tasks. Table 9 compares these algorithms’ performance on the EMBRACE dataset. The Bi-LSTM algorithm performs closest to our model but is still slightly lower in every metric. The learning vector quantization and random forest models perform moderately well but do not match the high performance of our EMBRACE framework.

**Table 9:** Comparisons of our proposed algorithm based on overall burnout prediction accuracy with state-of-the-art algorithm performance on our collected EMBRACE^a^ dataset. Data are presented as mean% (SD%).

Algorithms	Accuracy	Precision	Recall	*F*_1_-score
Learning vector quantization [67]	88.0 (1.1)	88.5 (0.9)	88.0 (1.1)	88.4 (0.8)
Random forest [66]	87.09 (1.0)	87.7 (0.9)	87.09 (1.0)	87.5 (1.1)
Bi-LSTM^b^ [61]	93.6 (0.8)	93.9 (0.6)	93.5 (0.9)	94.0 (0.7)
Ours	94.7 (0.9)	94.7 (0.5)	94.7 (0.9)	95.1 (0.2)

^a^EMBRACE: Explainable Multitask Burnout Prediction Using Adaptive Deep Learning.

^a^Bi-LSTM: bidirectional long short-term memory.

### Explainability Study

The primary focus of the explainability study in the EMBRACE framework is to enhance the clinical trustworthiness and usability of the burnout prediction system through an easily interpretable, explainable ML model. This study aims to make complex model predictions comprehensible to the end users (resident physicians and clinicians) by providing insights into how the predictions are derived, thus increasing their clinical utility.

#### Setup

We implemented the explainability module as a supplementary step in the EMBRACE system, focusing on two primary outputs: (1) the completion of a clinically validated burnout survey (Mini-Z) and (2) a summary of workplace activity, stress measures, and burnout indicators. The Mini-Z survey responses, which serve as a clinically explainable output, are automatically filled based on the model’s burnout prediction. These survey responses reflect the participants’ stress, workload, and overall satisfaction levels.

In this study, we adopted SHAP as our primary explainability tool for wearable sensor-based burnout and stress prediction. SHAP values assign importance scores to each feature used in the model, offering a detailed breakdown of how each feature contributes to the final prediction. These explanations are then converted into an intuitive format that can be easily interpreted by clinicians. For visualization, we generated 2 main outputs: SHAP value-based feature importance plots and a time-series summary of activities and stress indicators throughout the day.

#### Use of ML in Explainability

Our adaptive multitask deep learning model leverages time-series data from wearable sensors such as heart rate, EDA, and accelerometer readings to predict burnout. Once the predictions are made, we use SHAP to interpret the contributions of each sensor reading toward the burnout prediction. For example, SHAP values illustrate whether elevated heart rate or prolonged sedentary periods are significant contributors to burnout risk.

In addition to the burnout predictions, we also predict the responses to Mini-Z survey questions, which include satisfaction with work, perceived stress, and control over workload. SHAP analysis allows the model to break down these predictions, showing how different stressors (eg, EMR workload or workplace interruptions) influence the outcomes. This transparency ensures that clinicians can trust the model’s predictions and understand the underlying factors driving these outcomes.

#### Visualization

Visualization plays a crucial role in translating the explainable ML outcomes into actionable insights for clinicians. Our model outputs two primary visual aids:

1. Feature Importance Plot:
  The SHAP-based feature importance plot ranks the top features contributing to burnout, such as heart rate variability, sedentary activity duration, or frequent interruptions. Clinicians can use this ranking to quickly identify key stressors associated with burnout risk and focus on interventions for the most significant factors.
2. Activity and Stress Summary:
  This time-series summary visualizes the participant’s daily activity breakdown, including tasks such as writing notes, responding to emails, and attending meetings. These activities are mapped to stress levels measured by the wearable sensors. The summary offers clinicians an at-a-glance overview of how workday activities contribute to stress and burnout risks.

Below are sample tables that represent these visualizations for one participant (sample no. 1).

These tables provide clinicians with a clear understanding of key features influencing burnout (Table 10), a summary of daily activities (Table 11), and a summary of stress levels (Table 12). This visualization enables clinicians to take targeted actions based on the specific stressors and activities contributing to burnout.

**Table 10:** Feature importance table for person (sample no. 1).

Feature	SHAP^a^ value	Importance rank
Heart rate variability	0.45	1
Sedentary activity duration	0.38	2
Time spent writing notes	0.35	3
EMR^b^ time	0.30	4
Interruptions frequency	0.25	5
Sleep quality (night before)	0.20	6

^a^SHAP: Shapley Additive Explanations.

^b^EMR: electronic medical record.

**Table 11:** Activity summary table for person (sample no. 1).

Activity	Time spent (hours)	Percentage of the day
Writing notes	4.5	45
Responding to emails	2.0	20
Attending meetings or presenting	1.5	15
Searching for information	1.0	10
Breaks (away from keyboard)	1.0	10

**Table 12:** Stress summary table for person (sample no. 1).

Stress level	Duration (hours)	Percentage of the day
High stress	3.5	35
Medium stress	2.5	25
Low stress	3.0	30
Neutral or relaxed	1.0	10

#### End-of-Day Email Alerts and Feedback Collection

To ensure proactive interventions, the EMBRACE framework sends an end-of-day email to the resident physician with a summary of the day’s activities, stress levels, and a filled-out Mini-Z survey. The email includes a visual breakdown of the day’s workload and corresponding burnout predictions, along with recommendations to mitigate future burnout risks. Clinicians and residents can review the survey and workplace summary to identify stressors and consider adjustments in daily routines.

Furthermore, the system integrates a feedback loop, where physicians can provide input on the model’s predictions and explanations. The feedback is collected through a web-based form linked in the email, where clinicians can indicate whether the burnout prediction and activity summary matched their actual experience. This feedback is invaluable for further refining the EMBRACE model, ensuring it adapts to the unique experiences of individual residents and physicians over time.

By integrating SHAP values, visualization tools, and real-time feedback collection, the EMBRACE framework effectively bridges the gap between complex ML models and clinically actionable insights. The explainability study showcases how these tools enhance both the interpretability and usability of the burnout prediction system, enabling physicians to make informed decisions regarding their well-being.

#### Evaluation of the Satisfaction of Explainable Visualization

Additionally, we conducted an end-of-study survey to evaluate the impact of our visualizations on participants’ understanding of burnout. The survey, completed by 23 out of 28 participants, assessed the clarity of the 3 explanations: feature importance summary, activity summary, and stress summary. Among the 23 participants, 20 (87%) reported that the feature importance summary was the most impactful. Furthermore, 21 (91%) participants expressed high satisfaction with the explainability of the feature importance summary, 18 (78%) participants were highly satisfied with the activity summary, and 21 (91%) participants were highly satisfied with the stress summary explanation. These findings underscore the importance of explainability in promoting user trust and comprehension of predictive models in clinical settings. Table 13 provides the details of our end-of-study survey results.

**Table 13:** Poststudy survey responses: satisfaction with feature importance, activity summary, and stress summary explanations.

Satisfaction level	Feature importance (n=23), n (%)	Activity summary (n=23), n (%)	Stress summary (n=23), n (%)
Highly satisfied	20 (91)	18 (78)	21 (91)
Somehow satisfied	1 (4)	3 (13)	2 (9)
Neutral	0 (0)	1 (4)	0 (0)
Somehow dissatisfied	1 (4)	1 (4)	0 (0)
Totally unsatisfied	0 (0)	0 (0)	0 (0)

## Discussion

### Validation of EMBRACE With Wearable Sensors, MAML, and Correlation Analysis

Our proposed EMBRACE framework demonstrated that adaptive multitask deep learning, integrated with wearable sensor data and SHAP-based explanations, effectively predicts future burnout among resident physicians, significantly improving clinical interpretability, trust, and actionable insights.

We have chosen the Empatica E4 wearable sensor for its robust and validated capability to capture key physiological indicators associated with burnout, stress, and exhaustion, including heart rate, EDA, skin temperature, and accelerometry data. The device’s accuracy and widespread use in clinical research ensure reliable data collection, aligning with our objective to quantify predictors and confounders of burnout. Established studies have demonstrated strong correlations between heart rate and EDA with stress, anxiety, and exhaustion, making these metrics critical for identifying burnout-related patterns. Furthermore, the inclusion of skin temperature and accelerometry enriches the dataset by providing insights into thermoregulation and activity levels, which are important confounders for differentiating physical and psychological stressors.

We have used the MAML algorithm in this study because it is particularly suited for scenarios with limited labeled data and the need to generalize across diverse tasks, such as detecting burnout indicators across individuals with varying physiological baselines. Unlike traditional ML algorithms, MAML efficiently adapts to new tasks with minimal fine-tuning, enabling personalized predictions in dynamic and heterogeneous environments. Additionally, its meta-learning approach ensures robust model performance even when faced with variability in wearable sensor data, making it ideal for addressing the challenges of burnout prediction in real-world settings.

**Figure 3:**
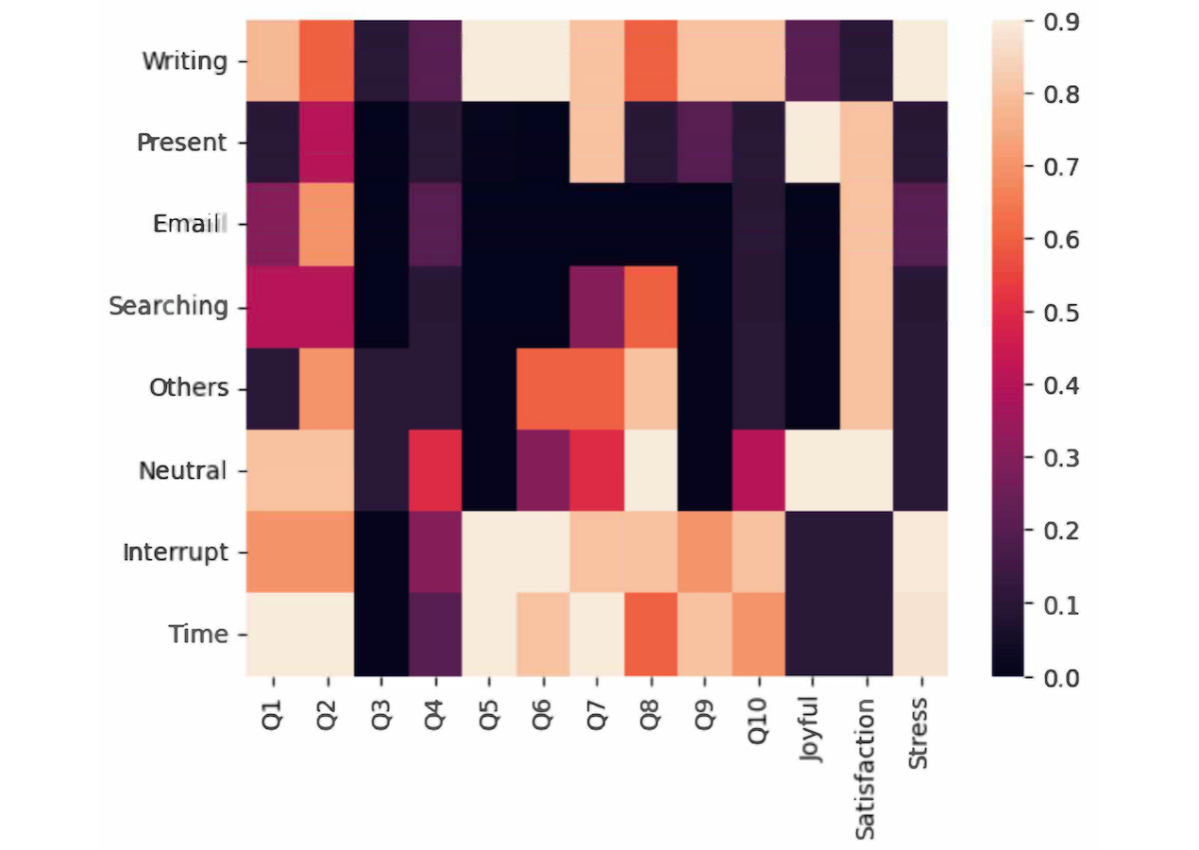
Pearson correlation coefficient (R) heatmap among detected workplace activities, stress levels, Mini-Z survey responses, and burnout measures.

The findings of this study provide valuable insights into the relationship between workplace activities, stress levels, and burnout among resident physicians. By applying the multitask workplace activity and stress detection algorithm to our collected dataset (D3), we effectively analyzed and predicted burnout levels with high accuracy. The correlation analysis using the Pearson correlation coefficient technique between predicted workplace activities, stress levels, Mini-Z questionnaire responses, and burnout measures offers a comprehensive view of the stress-burnout relationship. These correlations are visualized in Figure 3.

Our results reveal several key relationships. Foremost, highly interruptive and time-pressured workplace activities were strongly associated with elevated stress levels and negative responses to the Mini-Z questionnaire. These findings align with previous studies, which demonstrate that frequent interruptions and increased workload pressures contribute to burnout. For instance, residents who experience continuous interruptions may struggle to focus on critical tasks, leading to higher stress and dissatisfaction. This is evident in survey items such as Q5 (“I feel a great deal of stress because of my job”) and Q6 (“The amount of time I spend on the EMR at home”), both of which exhibited strong correlations with time-pressured activities.

Moreover, the correlation between note-writing activities, especially related to EMR documentation, and higher stress levels further underscores the role of administrative tasks as a significant contributor to burnout. Stress related to EMR use has been widely reported in health care literature, and our analysis corroborates these findings, confirming that documentation burdens are a key stressor for residents. As shown in the correlation heatmap, these tasks are closely aligned with burnout predictors.

Interestingly, a positive correlation between presentation activities and job satisfaction was observed. Activities that involve presenting or participating in discussions were linked to a more joyful work environment, suggesting that these tasks may foster a sense of professional accomplishment or engagement, serving as protective factors against burnout.

From an explainability perspective, the SHAP values were crucial in providing insights into how specific workplace activities and physiological measures influenced burnout predictions. Visualizing the contribution of individual features, such as heart rate variability and sedentary activity duration, enhanced clinical trust in the model’s predictions. The real-time interpretability facilitated by email alerts and feedback loops played a key role in engaging residents with their data, providing a feedback mechanism for continuous model improvement.

### Conclusion and Future Work

This paper introduces the EMBRACE framework, a novel multitask adaptive deep learning approach designed for predicting and explaining burnout in resident physicians. By integrating wearable sensor data with the clinically validated Mini-Z burnout survey, EMBRACE provides a unique approach to clinically explainable burnout prediction. The combination of workplace activity recognition, stress level detection, and explainable burnout prediction offers clinicians actionable insights into the burnout risks faced by resident physicians.

Our results demonstrate high prediction accuracy across all tasks, with the framework outperforming several baseline models, including Bi-LSTM, learning vector quantization, and random forest. The SHAP-based explainability mechanisms also significantly enhanced the interpretability of model outputs, building clinician trust and enabling real-time interventions based on predicted burnout risks.

Despite these promising findings, the study has limitations. The relatively small sample size of 28 participants limits the generalizability of the results. Further studies with larger, more diverse populations are needed to validate the findings. Additionally, while EMBRACE offers detailed insights into stress and burnout, further research is required to assess the long-term effectiveness of the suggested intervention strategies. A longitudinal satisfaction study would also be valuable in evaluating the impact of explainable AI in reducing burnout in clinical settings.

Future work will focus on expanding the framework by incorporating additional physiological and behavioral metrics, such as sleep quality and social interactions, to provide a more comprehensive assessment of burnout risks. We also aim to develop personalized interventions based on real-time predictions, allowing for tailored strategies to mitigate burnout before it escalates. Scaling the framework to different clinical environments and assessing its adaptability in various health care settings will also be key areas of exploration.

## Appendix

Multimedia Appendix 1 Description of the study’s dataset.

## Abbreviations

AI: artificial intelligence
Bi-LSTM: bidirectional long short-term memory
CNN: convolutional neural network
DeepLIFT: Deep Learning Important Features
EDA: electrodermal activity
EMBRACE: Explainable Multitask Burnout Prediction Using Adaptive Deep Learning
EMR: electronic medical record
HIPAA: Health Insurance Portability and Accountability Act
LIME: local interpretable model-agnostic explanations
LOOCV: leave-one-out cross-validation
LSTM: long short-term memory
MAML: Model-Agnostic Meta-Learning
ML: machine learning
MLP: multilayer perceptron
MTL: multitask learning
NASA-TLX: National Aeronautics and Space Administration Task Load Index
PGY: postgraduate year
PSS: Perceived Stress Scale
RBF: radial basis function
RSME: Rating Scale Mental Effort
SAM: Self-Assessment Manikin
SHAP: Shapley Additive Explanations
SWELL-KW: SWELL Knowledge Work
WESAD: Wearable Stress and Affect Detection
