# Supplementary material for: Explainable Multitask Burnout Prediction Using Adaptive Deep Learning (EMBRACE) for Resident Physicians: Algorithm Development and Validation Study
Source: JMIR AI. 2026 Jan 8;5:e57025. doi: 10.2196/57025 (PMC12828314; doi:10.2196/57025)
Supplement: Multimedia Appendix 1 [file ai_v5i1e57025_app1.docx]

## Publicly Available Wearable Stress and Affect Detection Dataset (D1)

Upon device synchronization and proper placement on the body, each participant underwent a laboratory protocol divided into several distinct phases:

- - **Baseline phase**: The participants were instructed to sit or stand in a neutral position at a table and read magazines for 20 minutes.
  - **Amusement phase**: The participants watched humorous videos for a duration of 6 minutes.
  - **Stress phase**: Induced by the Trier Social Stress Test [49], this phase lasted 10 minutes and involved a public speaking task and a mental arithmetic challenge. Participants were required to speak about their personal strengths and weaknesses in front of a mock human resources panel, followed by a task of serial subtraction (subtracting 17 from 2023 continuously without making errors).
  - **Meditation phase**: A guided breathing exercise session with eyes closed, led by an expert, to help participants return to a neutral affective state.
  - **Recovery phase**: Following the meditation phase, both devices were resynchronized and subsequently removed.

## Publicly Available Stress and User Modeling Dataset, SWELL-KW Dataset (D2)

The SWELL-KW dataset is a comprehensive collection of sensor data and subjective stress assessments gathered from participants engaged in workplace activities [50, 51, 52]. This dataset includes accelerometer, heart rate, and galvanic skin response (GSR) data, along with activity labels and stress assessments from four clinically validated survey tools.

*Study Procedure:* The SWELL-KW dataset was collected from 25 participants (8 females and 17 males), comprising both males and females, with an average age of 29 years and a standard deviation of 4.2 years [50, 51, 52]. The study aimed to evaluate stress levels induced by common workplace stressors, such as email interruptions and time pressure. Participants performed various workplace activities over a 3-hour session, during which sensor data were continuously recorded. The sensors used include accelerometers, heart rate monitors, and GSR sensors. The accelerometers operated at a frequency of 50 Hz, heart rate monitors at 1 Hz, and GSR sensors at 10 Hz. Data were stored securely and shared in a de-identified format to ensure participant privacy and facilitate further research.

They manipulated working conditions to assess stress responses among participants by creating three scenarios: a neutral condition where participants worked without time constraints (up to 45 minutes), a “Time Pressure” condition where the time was reduced to 2/3 of the neutral time (maximum 30 minutes), and an “Interruptions” condition where participants received eight emails during the task, varying in relevance and response requirements. All 25 participants experienced these conditions in a counterbalanced order, starting with the neutral condition to establish a baseline. Participants performed knowledge-based tasks, such as writing reports and creating presentations on predefined topics, in a controlled lab environment, simulating realistic office work by allowing internet research and requiring interaction with incoming emails. To ensure serious engagement, participants were informed that they needed to complete all tasks to receive full compensation and might have to present their work. The experiment spanned approximately three hours, with each of the three stressor conditions taking about an hour, including relaxation phases and breaks. Participants were instructed to avoid smoking or caffeine before the experiment, signed consent forms, and were debriefed afterward. The procedure was designed to accurately capture stress responses induced by the experimental conditions.

*Activities:* The dataset includes labeled activity data for several activities: make presentation, paper writing, paper plan, write email, read email, program, make overview, search information, and away from keyboard. Activities were annotated using video recordings, with labels assigned based on the observed activities. The detailed annotation process ensured high accuracy and reliability in the activity labels, providing a rich source of contextual information for analyzing stress patterns.

*Surveys Ground Truths:* Four validated surveys were used to assess participants’ subjective experiences: the NASA Task Load Index (NASA-TLX), the Rating Scale Mental Effort (RSME), the Self-Assessment Manikin (SAM), and the Perceived Stress Scale (PSS).

1. **NASA-TLX:** This survey consists of six questions measuring mental, physical, and temporal demand, performance, effort, and frustration on a 21-point scale. Stress levels are determined by averaging the scores across the six dimensions, with higher scores indicating higher perceived task load and stress.
2. **RSME:** A single-item scale where participants rate their mental effort on a continuous scale from 0 (no effort) to 150 (extreme effort). The continuous scale score is used directly, with higher scores indicating higher mental effort and stress.
3. **SAM:** Measures three dimensions of emotion (valence, arousal, dominance) using pictorial scales with scores ranging from 1 to 9 for each dimension. Stress levels are derived by analyzing the scores on the valence, arousal, and dominance dimensions, with higher arousal and lower valence scores indicating higher stress.
4. **PSS:** A 10-item questionnaire assessing how unpredictable, uncontrollable, and overloaded respondents find their lives, with scores ranging from 0 to 40. A 10-item questionnaire assessing how unpredictable, uncontrollable, and overloaded respondents find their lives, with scores ranging from 0 to 40. This scale provides a global measure of perceived stress.

## Algorithm

**Algorithm S1.** Multitask Few-Shot Deep Domain Adaptation with Model-Agnostic Meta-Learning (MAML)

# 1: Initialization:

2: Initialize source model parameters *W_source_*.

3: Define source domain tasks *T_source_* as *T_act_* and *T_stress_*.

# 4: Source Domain Training:

5: Train the model on the source domain tasks *T_source_* using multitask deep learning.

6: Update source model parameters *W_source_* using gradient descent.

# 7: Few-Shot Adaptation:

8: Select 10 labeled samples from each class *l_target_* from the target domain tasks *T_target_*, specifically *Tsurvey answers* and *Tburnout*.

9: Define a new task *t_target_* in the target domain with the labeled samples *l_target_*.

10: Clone the source model and initialize cloned model parameters *W_target_* with source model parameters *W_source_*.

11: **for** each target domain task *t_target_* **do**

# 12: Inner Loop Training:

13: Perform a few gradient update steps on the cloned model *W_target_* using labeled samples *l_target_* from the target domain task *t_target_*.

14: Compute task-specific loss *L_target_* and update cloned model parameters *W_target_* using gradient descent.

# 15: Outer Loop Update:

16: Compute gradient of task-specific loss *L_target_* with respect to initial parameters *W_source_* of the source model.

17: Update initial parameters *W_source_* of the source model using the gradient.

18: **end for**

19: **Evaluation:**

20: Evaluate the adapted model on target domain tasks *T_target_* by predicting Mini-Z survey questionnaire answers.

21: Estimate the overall burnout scale class based on predicted answers.

## Equations

*R*^2^ loss can be represented as follows:

$R^{2}=1- \frac{MeanSquaredError}{Variance\left( y \right)}$ (S1)

Percentage of *R*^2^ loss can be represented as follows:

${Percentage R}^{2}=100*(1- \frac{MeanSquaredError}{Variance\left( y \right)})$ (S2)

Balance accuracy formula is as follows

$Balanced Accuracy= \frac{1}{T}\sum_{t=1}^{T} Accuracy(t)$ (S3)

where *T* is the total number of tasks, Accuracy(*t*) is the accuracy of the *t*th task, calculated as

Accuracy(*t*) = $\frac{True Positives \left( t \right)+True Negatives (t)}{Total Instances (t)}$ (S4)

## Table S1. Descriptive Characteristics of the EMBRACE dataset.

| **Variable** | **Description** |
| --- | --- |
| **Study Design** | Prospective observational study. |
| **Institution** | Renowned teaching-based medical center. |
| **Sample Size** | 28 internal medicine resident physicians. |
| **Age (Years)** | Mean: 27.5, SD: 3.5. |
| **Sex Distribution** | Female: 15 (53.6%), Male: 13 (46.4%). |
| **Postgraduate Year (PGY)** | PGY1: 10 (35.7%), PGY2: 9 (32.1%), PGY3: 9 (32.1%). |
| **Marital Status** | Married: 12 (42.9%), Not Married: 16 (57.1%). |
| **Inclusion Criteria** | Actively engaged in the residency program. |
| **Exclusion Criteria** | Medical conditions interfering with stress and burnout assessment. |
| **Data Collection Period** | Continuous monitoring during daily duties, spanning 2 to 7 days per participant. |
| **Total Observation Days** | 98 days. |
| **Work Hours Per Day** | 8–13 hours per day, Mean: 10.5 hours/day. |
| **Total Physiological Data** | Approx. 1,029 hours of physiological data. |
| **Ground Truth Data** | 98 daily surveys. |
| **Burnout Days** | 33 days, involving 19 residents. |
| **Wearable Device Used** | Empatica E4 wristband. |
| **Outcome Measures** | Daily burnout status based on surveys. |
